# Science for implementation: the roles, experiences, and perceptions of practitioners involved in the Intergovernmental Panel on Climate Change

**DOI:** 10.1007/s44168-022-00025-2

**Published:** 2022-09-24

**Authors:** M. A. North, N. B. Hunter, D. C. Roberts, R. Slotow

**Affiliations:** 1 School of Life Sciences, University of KwaZulu-Natal, Private Bag X54001, Durban, 4000 South Africa; 2 Sustainable and Resilient City Initiatives Unit, eThekwini Municipality, Durban, South Africa; 3 Department of Genetics, Evolution and Environment, University College, London, UK

**Keywords:** Climate Change, IPCC, Science-policy interface, Pracademic, Practitioner, Climate-change policy

## Abstract

In the climate change arena, the assessments of the Intergovernmental Panel on Climate Change (IPCC), while not policy prescriptive, are important for informing international negotiations and the decisions of policy and practice communities. Since 2015, when the Paris Agreement was adopted, there has been an increasing demand for climate action and calls for the inclusion of a broader base of stakeholders in global assessments. We use publicly available information on IPCC authors, and the results of a survey (*n* = 29) and interviews (*n* = 17) with authors who apply science in practice (practitioners), to examine their roles, experiences, and challenges in the IPCC’s Fifth Assessment. The results show that while practitioners form only a very small proportion of authors, they contribute real-world experience, including the translation of academic language into meaningful messages for decision-makers. Although familiar with working at the interface of science and practice, study participants described barriers to their participation and shared several recommendations for improving the relevance, uptake, and timeliness of IPCC assessments, and practitioner participation. Our findings indicate that IPCC assessments provide an evidence base to support action on climate change, and due to their skill set, practitioners are often able to make contributions that allow for greater accessibility by decision-makers.

**Graphical Abstract:**

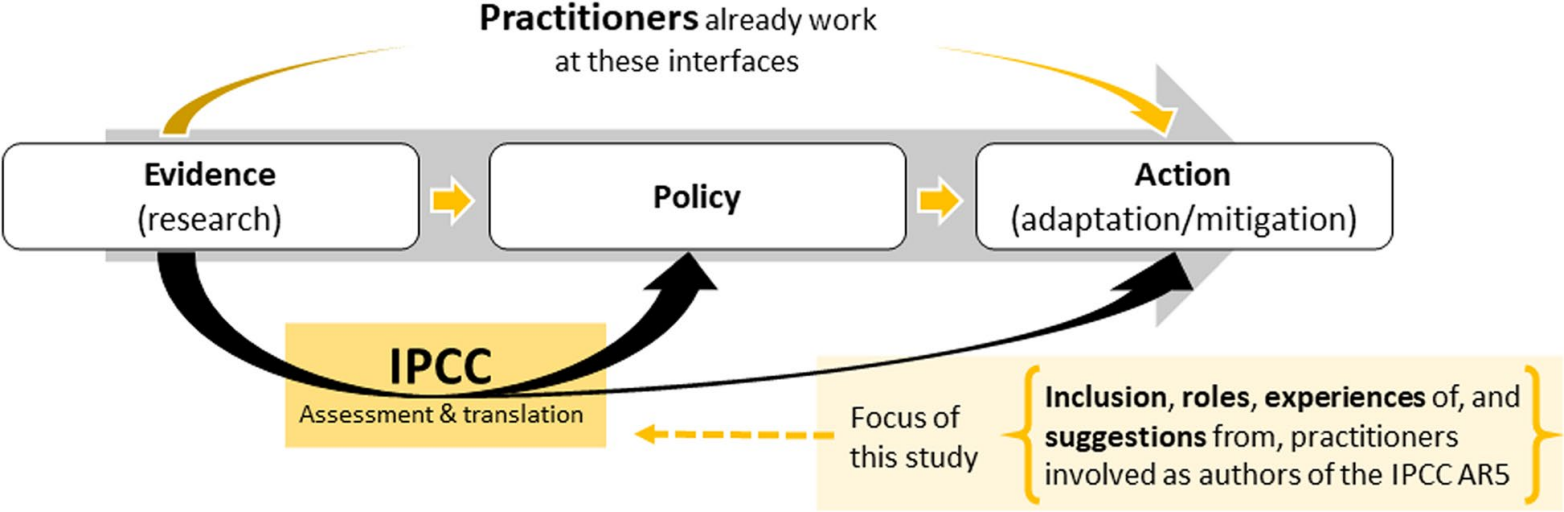

**Supplementary Information:**

The online version contains supplementary material available at 10.1007/s44168-022-00025-2.

## Introduction

Never before has there been a time of such heightened climate change awareness, with increasing social mobilization and calls for action (Molina and Abadal 2021). The Intergovernmental Panel on Climate Change (IPCC), as the international body responsible for assessing the state of knowledge of human-induced climate change, not only provides the most up-to-date information on the physical, natural, and social science behind climate change (IPCC 2021a), but also highlights possible solutions (Howarth et al. 2017; IPCC Secretariat 2017a, b). Currently approaching the end of its sixth assessment, the previous Fifth Assessment Report informed the negotiation of the Paris Agreement (Ourbak and Tubiana 2017). This international agreement, adopted by the United Nations Framework Convention on Climate Change (UNFCCC) in December 2015 (United Nations 2015), acknowledges the importance of a range of actors “including those of civil society, the private sector, financial institutions, cities, and other subnational authorities” in realizing global climate change goals (Roberts 2016, 3-4).

As key actors for the implementation of climate change responses, practitioners provide an important perspective when assessing climate change impacts and solutions. Defined as “skilled professionals actively engaged in the development and application of practical responses to global challenges” (Howarth et al. 2017, 3), practitioners are employed by institutions focused on the application of knowledge and include professionals such as policymakers, decision-makers, engineers, and investors (Viner and Howarth 2014). Practitioners can have both a local and global presence and work on widely varied projects across disciplines and sectors (Howarth et al. 2017).

IPCC reports are authored by thousands of volunteer scientists, who are nominated by their government focal points, accredited observer organizations, or members of the IPCC Bureau, which also oversees author selection based on scientific expertise and regional representation (Agrawala 1998; Ho-Lem et al. 2011; IPCC 2019). All IPCC authors, regardless of background, work context, or experience, are called to assess “the state of scientific, technical, and socio-economic knowledge on climate change; its impacts and risks; and response options” (IPCC 2019, 1). This is traditionally an academic practice, and unsurprisingly, the leadership and authorship of these reports are dominated by academics and researchers (Howarth and Monasterolo 2017; Viner and Howarth 2014). While the IPCC does not undertake research, and is explicitly policy-relevant and not policy prescriptive, the IPCC procedures highlight “broad, balanced participation in the author teams” (IPCC Secretariat 2010, 1), with the involvement of industry experts being a clear priority of the current IPCC Chair, together with a stronger focus on solutions (Howarth et al. 2017; Schiermeier and Tollefson 2015; Yamineva 2017). Considering that practitioners are frequently the main end users of assessment reports, their input into the process is crucial to ensure that content is relevant to the needs of its users (Gordon et al. 2014; Howarth et al. 2017; Viner and Howarth 2014), and perceived to be legitimate (Clark et al. 2002). By working together, practitioners and academics are better able to ensure that IPCC reports are relevant to and accessible by all intended users, including decision-makers in public policy (Gustafsson and Lidskog 2018; Viner and Howarth 2014)—one critical component of climate action.

The issues of relevance (also referred to as salience), credibility, and legitimacy have long been discussed in the context of the IPCC (Cash et al. 2003; Clark et al. 2002; Vardy et al. 2017), with some scholars highlighting a link between the diversity of climate change researchers and authors of these reports and the appropriateness of the content for the context-specific needs of decision-makers (Ho-Lem et al. 2011; Pasgaard and Strange 2013; Vardy et al. 2017). Previous research on the IPCC has highlighted issues of regional imbalance, with most IPCC authors coming from countries in the Global North and few from the most vulnerable countries or where English is not commonly used (Corbera et al. 2015; Pasgaard et al. 2015). Other research examined issues of gender (Corbera et al. 2015; Nhamo and Nhamo 2018) and disciplinary representation (Bjurström and Polk 2011; Callaghan et al. 2020), as well as the privileging of some forms of knowledge, leading to disciplinary biases (Beck et al. 2014; Hughes 2015; Obermeister 2017), the dominance of certain professions (Devès et al. 2017; Victor 2015), and the marginalization of indigenous knowledge (Ford et al. 2016). The IPCC’s efforts to address these shortcomings have improved Global South participation (Ho-Lem et al. 2011; Okereke 2017), gender, and regional representation, yet some improvements in diversity lag behind, particularly within the working group on the physical science basis of climate change (WGI) and with regard to author seniority (Standring and Lidskog 2021).

There is broad agreement that the IPCC’s constructions of climate change are shaped by both science and politics (Beck 2011; Hughes 2015; Okereke 2017; Siebenhüner 2002; Skodvin 2000). Corbera et al. (2015) show that the social conventions and processes for selecting authors and recognizing authoritative knowledge benefit particular institutions and collaborations and are tied to political and economic power. The authors who have previously participated in IPCC assessments are a source of authority on how the assessment is conducted. This experience is beneficial, serving to bridge assessments (Venturini et al. 2020); however, it also places these authors at an advantage over first-time authors, giving them more influence over report content (Hughes and Paterson 2017).

Nevertheless, the contribution of practitioners to previous IPCC reports is unclear, with the scholarship on IPCC authors outlined earlier not distinguishing between academics/researchers and practitioners. As such, the objectives of this research were to understand what practitioners brought to IPCC assessments: to what extent they are included, their specific roles in the process, and their experiences, including what challenges they faced, as well as their suggestions for improving practitioner involvement and, thereby, the relevance of IPCC reports for decision-makers. We surveyed and interviewed practitioners involved as authors of the IPCC’s Fifth Assessment Report (AR5) to investigate these issues. We also examined the list of authors of the Sixth Assessment (AR6) to obtain insight into the changes in practitioner participation over time.

In this article, we begin by describing governance at the science-policy interface, followed by an elucidation of literature on the relationship between scientific evidence and policymaking and how this relates to authorship in the IPCC. We then describe our methods and the specific challenges of this research; present our results, starting with a representation of practitioners in AR5, broken down by region, gender, and leadership roles within the process; and compare this representation to that of AR6, to see whether there have been any changes over time. We then discuss the results of a survey of practitioner authors of AR5, including their perception of challenges they might have faced during their participation, and present the results of interviews of a subset of these practitioners, highlighting the themes identified during the interviews. Finally, we present a set of suggestions from the survey respondents and interviewees, which serves as a launchpad for the discussion, where we pull the findings of this study together in the context of the wider literature and end off with a set of recommendations to improve the uptake and implementation of IPCC reports.

## Scientific evidence and policymaking

Scientific panels like the IPCC serve as boundary organizations (Gustafsson and Lidskog 2018; Haas 2017), working at the interface between science and politics, and facilitating the flow of information between public policy and research communities to meet the need for evidence-based policy and practice (Hotes and Opgenoorth 2014; Howarth and Painter 2016; Morin et al. 2017; SESYNC 2012). While not all authors agree that this term should be applied to the IPCC (Hughes and Vadrot 2019; Hughes and Paterson 2017), we find it relevant since the IPCC fulfills a key political function in certifying knowledge that can serve as a foundation for policy and inform societal responses to problems of unsustainability (Beck et al. 2014; Cornell et al. 2013; van der Sluijs et al. 2010).

Global research programs and assessments have provided scientific evidence for the existence of global warming and other environmental challenges and have significantly influenced environmental negotiations (Haas 2017). However, despite the involvement of both the scientific and policy communities in the formulation and revision of IPCC reports (De Pryck 2021), this evidence has yet to translate into comprehensive solutions (Beck and Mahony 2018). Translation of scientific (and other) evidence into policy is complex and often leads to frustrations on both sides, “because clearly presented and robust evidence does not always have the desired effect on policy processes” (Strydom et al. 2010, 2). Policymakers’ decisions and actions are, to a large extent, made in response to social pressures, political values, and ideologies (Leuz 2018), rather than scientific evidence that may not meet immediate policy needs, or may not be framed in a useable way (Haas 2017).

Group diversity—of knowledge systems, disciplines, experience—is key for boundary organizations (Cornell et al. 2013), and this has implications for who participates in the work of these bodies. Scientific knowledge is recognized to be necessary—but not sufficient—for developing relevant and viable policies, with experts’ range of experience and affiliations also important (Beck et al. 2014).

One of the many factors influencing the decision-making process is the “extent to which policymakers and scientists attempt to understand each other’s viewpoints” (Strydom et al. 2010, 2). As such, the ability to effectively communicate research for policy audiences requires a deliberate effort to convey the message accurately across different worldviews and with an understanding of the contexts and timelines within which policymakers work (Strydom et al. 2010), to “put scientific information into context and in proportion, using language that can be readily understood by policy-makers and other stakeholders” (Holmes and Savgård 2009, 715). A particular group of experts, known as pracademics, or boundary spanners (Posner 2009), are uniquely able to fill this role, bringing together valuable experience from both the practitioner and academic communities.

While there are numerous obstacles to the implementation of recommendations from these assessments, including a lack of political will, and opposing economic forces (van den Hove and Chabason 2009), ensuring that IPCC assessments are written in a way that meets the needs of the end users addresses one possible barrier. Considering and addressing the different barriers to implementation exemplifies a shift in the mandate of science, a rethinking of the role of science in global policymaking and, some argue, the need for new forms of boundary work (Beck and Mahony 2018; Cornell et al. 2013). If boundary work does change in this way, the role of practitioners, whose very work is to solve problems, may be of increasing importance. Calls for better integration of scientific and policy communities, and their stakeholders (Cornell et al. 2013; Gustafsson and Lidskog 2018), as well as the Paris Agreement’s call for a broader range of participants in addressing climate change (United Nations 2015) highlight the relevance of this study for organizations working at this science-policy interface.

## Methods

To assess the inclusion of practitioners as authors of IPCC reports, we used publicly available information on the authors of AR5 and AR6 (as of 2021/02/03) (IPCC 2021b). We then surveyed and interviewed a subset of practitioner authors from AR5 to ascertain their specific roles in the process and their experiences, including any challenges they may have faced, as well as recommendations for improving practitioner involvement in the IPCC.

**Fig. 1:**
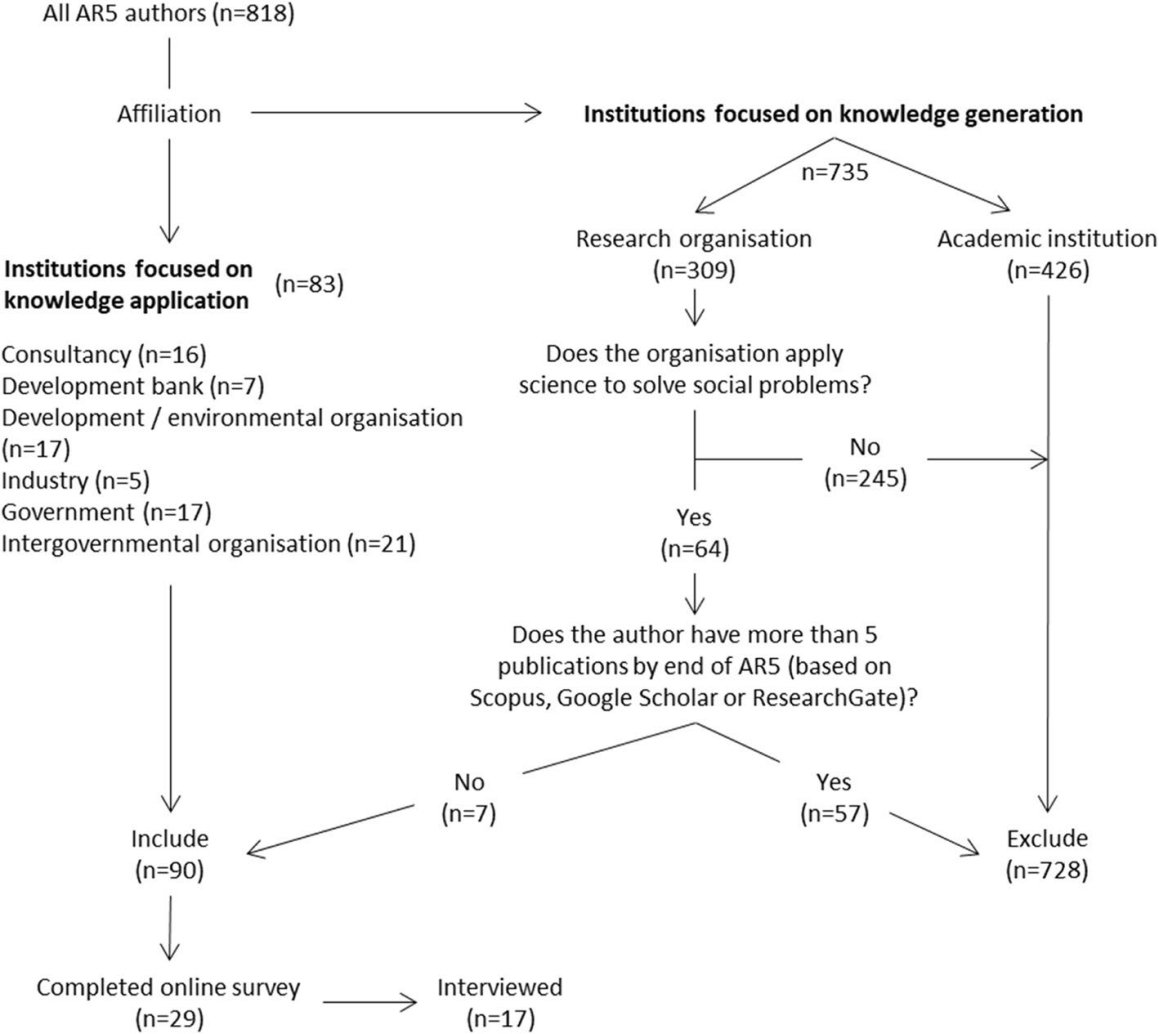
Decision pathway for determining which authors from the Fifth Assessment Cycle (AR5) of the Intergovernmental Panel on Climate Change were contacted about their experience as practitioners. The institutions listed as “focused on knowledge application” correspond with examples listed by Viner and Howarth (2014) (“professionals such as policymakers, decision-makers, engineers, and investors”), with the five institutions listed as “industry” including multinational engineering corporations and reinsurance and energy companies

The authors of AR5 and AR6 were coded as practitioners based on their affiliation as it is reported on the IPCC website (IPCC 2021b): whether the institution was primarily involved in knowledge application or generation (see below and Fig. 1). We followed a multistage selection process to determine eligible participants (Fig. 1): First, authors at academic institutions, engaged in knowledge generation, were excluded, while those working at institutions that focus on the application of knowledge for profit, or on solving societal problems, such as different levels of government, consultancies, or development banks, were designated as practitioners and included. We then assessed the eligibility of individuals working at research organizations, including parastatals (e.g., national meteorological agencies) that apply science to social problems, based on their publication history as reported by Web of Science, Scopus, and/or Google Scholar. Individuals working at such institutions, who had published fewer than five peer-reviewed papers by the end of AR5 (2014), were included. Only authors working at research organizations during AR5 were excluded as practitioners based on their publication history.

It was not always straightforward identifying practitioners—for example, one respondent self-identified as a practitioner since this was their current role, even though further interrogation revealed that their primary affiliation at the time of authoring was with an academic institution. Consequently, the interview and survey results for this respondent had to be removed from our final dataset. This challenge has been raised in the discussion as an opportunity for future research.

Ninety of the 818 AR5 authors were classified as practitioners based on their employer at the time of authoring. Extensive efforts were made to track down publicly available up-to-date contact information on the Internet, which was available for only seventy-two authors. These authors were then contacted via email, which included a description of the study, confirmation of ethics approval (University of KwaZulu-Natal Humanities and Social Sciences Research Ethics Committee Protocol reference number HSS/0833/018), and request to complete an anonymous online survey (SurveyMonkey, USA). This was completed in 2018, initially focusing on African practitioners only. However, response rates were low, and the small sample size weakened any conclusions. Consequently, we decided to also survey non-African practitioners, to make this a global study. In 2021, the same survey was sent to all practitioner authors who had not yet participated (using Google Forms, USA) (see Additional file 1). The survey questions were designed to record respondents’ background information (demographics, education, and occupation), experience with the IPCC process, and any additional issues. Twenty-nine authors completed the survey, and 17 of these respondents agreed to undertake interviews (see Additional file 2 for further details).

Permission was received to record interviews which were then transcribed. During an initial reading of the transcripts, themes were identified in terms of roles/contributions, experiences, and challenges. Next, attention was paid to distinguishing between information specifically applicable to practitioners and to all IPCC authors, with only the former included. Finally, to gain a sense of how widely each theme was applicable, interviewees for whom individual themes were relevant were identified.

AR5 was a noteworthy assessment for several reasons. Approximately three-quarters (73%) of the authors were first-time authors (Hughes and Paterson 2017, 10), and substantially more publications were cited than previously (Mach et al. 2017). Moreover, AR5 findings were released a few months ahead of the twenty-first Conference of the Parties (COP21) of the UNFCCC and informed the negotiation of the Paris Agreement (IPCC Secretariat 2017a). Finally, we focused on AR5 because it is the most recent assessment that can be easily studied—approval is required from the IPCC to study authors of ongoing assessments, such as AR6, during which this research took place.

## Results

### Representation of practitioners in AR5

Of the 818 authors of AR5, 728 worked primarily in the generation of knowledge—mainly at academic institutions (426) and research organizations (302). The remaining 90 (11%) were employed at consultancies, development banks, for-profit companies, government, and intergovernmental or development/environmental organizations, focused on the implementation of knowledge.

Two-thirds (69%) of the 817 authors for whom country information was available were from countries in the Global North, and 78% were men, with Global South and women authors poorly represented (Table 1). Practitioners made up a greater proportion of authors from the Global South than North, with a very high percentage from Africa compared to other regions. working group 1 (WG1) had the smallest proportion of practitioners as authors (2%, *n* = 6), followed by WG2 (13%, *n* = 39) and WG3 (17%, *n* = 45) (see Additional file 2: Table S1). The different working groups also differed in their inclusion of authors from different regions, with WG2 and WG3 including more diversity. Leadership roles, particularly coordinating lead authors (CLAs), were predominately filled by authors involved more in knowledge generation than practitioners.

**Table 1:** Diversity of the authors of the Intergovernmental Panel on Climate Change’s Fifth Assessment Report, by Global North or South, region (*n* = 817), gender (*n* = 818), roles in the IPCC (*n* = 818), and practitioner status

	Practitioner		Others		Total	
	*n*	%	*n*	%	*n*	%
Socio-economic division^a^						
North	32	*6%*	528	*94%*	560	*69%*
South	57	*22%*	200	*78%*	257	*31%*
Region^a^						
Africa	31	*43%*	41	*57%*	72	*9%*
Asia	14	*10%*	129	*90%*	143	*17%*
Europe	16	*6%*	262	*94%*	278	*34%*
Latin America and the Caribbean	9	*12%*	67	*88%*	76	*9%*
North America	12	*6%*	182	*94%*	194	*24%*
Oceania	7	*13%*	47	*87%*	54	*7%*
Gender						
Women	24	*13%*	154	*87%*	178	*22%*
Men	66	*10%*	574	*90%*	640	*78%*
Role						
Coordinating lead author (CLA)	11	*9%*	115	*91%*	126	*15%*
Lead author (LA)	61	*11%*	483	*89%*	544	*67%*
Review editor (RE)	18	*12%*	130	*88%*	148	*18%*
**Grand total**	**90**	***11%***	**728**	***89%***	**818**	***100%***

^a^Country-level information was not available for one author

### Changes in representation in AR6

Despite the IPCC Chair prioritizing improved representation of authors from different regions and practitioners in AR6 (IPCC Secretariat 2017a), analysis of AR6 authors does not reveal substantial changes from AR5 (Additional file 2: Tables S2 and S3 and Fig. S1). Women are marginally better represented in AR6, making up a third of all authors; however, changes in the representation of regions and practitioners are small and varied. The representation of practitioners in chapter team leadership did not change between AR5 and AR6; however, in AR6, a greater proportion of review editors were practitioners.

### Survey results

The 29 survey respondents represented diverse genders, nationalities, language abilities, disciplines, and sectors, and all but one had postgraduate education. Respondents had between 8 and 42 years of experience in their field and had been variously involved in all aspects of the IPCC process: from technical administration (two served as chapter scientists (CSs)), authoring (eight contributing authors (CAs), 25 lead authors (LAs), and five coordinating lead authors (CLAs)), reviewing or editing the reports (11 expert reviewers (ERs) and eight review editors (REs)) to levels of management in the IPCC, including a WG co-chair and WG vice-chair (further detail on this background information may be found in Additional file 2: Table S5).

Most respondents (18) ranked scientific expertise as being the most important for authoring IPCC reports. Regional representation and policy experience also ranked highly, followed by gender equality, with ethnic diversity the least important. Authors from academic institutions were perceived to have the highest influence over content, followed by public sector authors, with the least influence by private sector authors.

Survey participants were asked to rate how strongly they agreed or disagreed with statements about the IPCC process: seventeen framed positively and eleven negatively. While most reported an overall positive experience working with the IPCC (27), responses to positively and negatively framed statements highlighted specific issues.

**Fig. 2:**
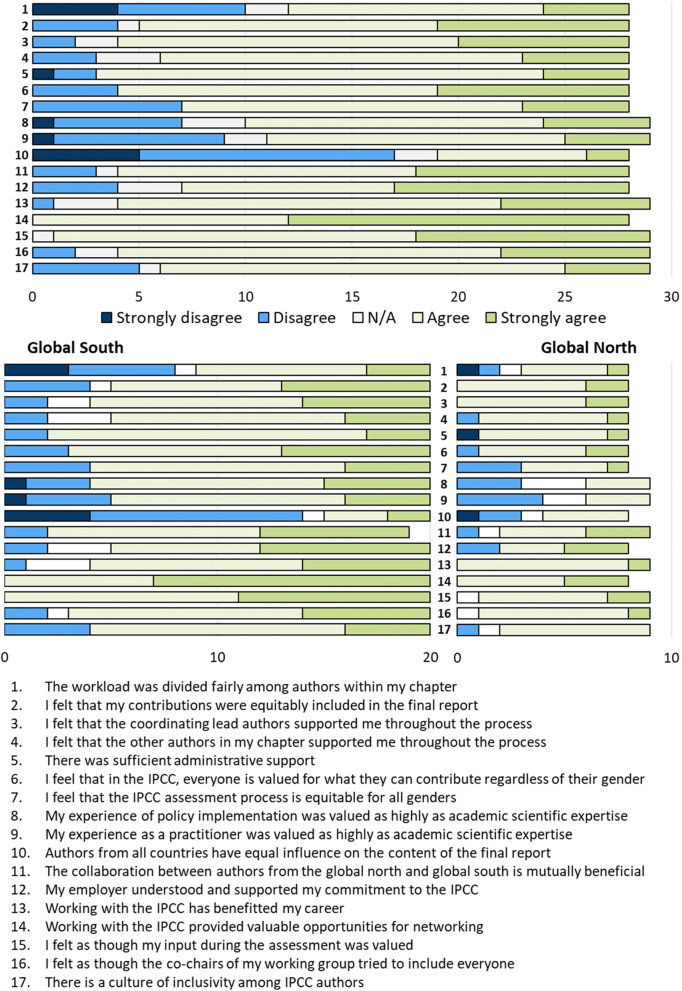
Participants’ responses to positively framed statements about aspects of the IPCC process, all together (top) and separated by respondents from the Global North or South (lower two). N/A = not applicable. Blue-shaded bars reflect the disagreement with the positive statements and thus highlight issues perceived by the respondents

Almost all respondents agreed with the positively framed statements (Fig. 2), with the only notable exception of the statement that “authors from all countries have equal influence over the content of the final report,” with 17 respondents disagreeing (61%; the survey did not investigate which countries had most influence). Interviewees who provided a reason for this pointed to chapter discussions taking place in a language that was not the mother tongue of Global South authors, or because they perceived that Global South authors did not have as much time to spend on IPCC work as those from the Global North.

**Fig. 3:**
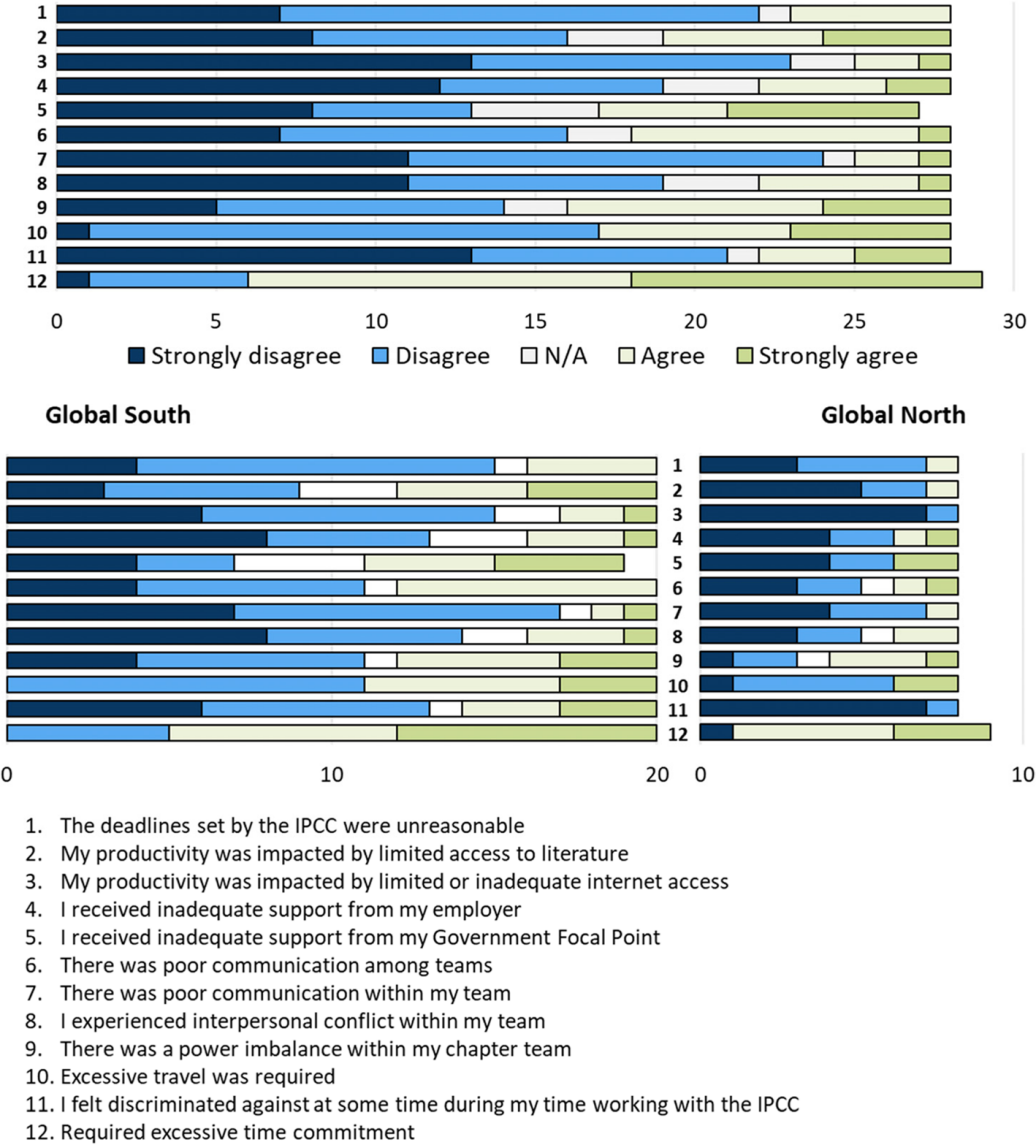
Participants’ responses to negatively framed statements about aspects of the IPCC process, all together and separated by respondents from the Global North or South. N/A = not applicable. Green-shaded bars reflect agreement with the negative statements and thus highlight issues perceived by the respondents

Negatively framed statements captured a more mixed message (Fig. 3). Most participants felt the assessment required excessive time commitment (23; 79%). Six respondents—all from the Global South—felt discriminated against during their time working with the IPCC. The phrasing of the statement did not allow interrogation into the nature of the discrimination; however, one of these respondents who was interviewed described discrimination against Global South countries because there was often no published evidence to support their experience, meaning that relevant issues could not be included (interviewee #2).

### Interview results

Seven female and 10 male practitioners were interviewed, seven from the Global South and 10 from the Global North, based on citizenship (Additional file 2: Tables S7 and S8). Over half of the interviewees were full-time consultants (one part-time in addition to being an unpaid research fellow—#13), with three working for intergovernmental organizations and four for NGOs. Two worked for the government, two for development banks, and one for a private company. Their work varied widely, from being somewhat like academic research (e.g., synthesis and analysis by consultants) to completely different (i.e., day-to-day work by the government employee or the private company CEO). Other interviewees juggled two roles simultaneously: intergovernmental organization and NGO (interviewee #8) or NGO and a consultant (#9). Some switched roles during the AR5 assessment: for example, from working at an NGO to working as a consultant (#10), or from consultant, to government, and back to consultant (#15).

The practitioner authors interviewed were highly educated—most (12) had PhDs, while three had master’s degrees—and had an understanding of, and familiarity with, the academic skills of critical assessment and synthesis. The practitioners interviewed as part of this study, represented a distinct subset of practitioners generally, with more-than-typical experience with this skill set, and may be better classed as pracademics.

All but two (#7 and #13) were full-time practitioners, with two having shifted from full- (#2) or part-time academic (#11) to full practitioner-type work. Additional file 2: Table S8 in the shows that many of those interviewed had published extensively. Only two conducted work with no “academic” element (#4, #14). Seven of the interviewees had prior IPCC author experience, dating back to AR2, with the remainder being first-time IPCC authors; however, two had had prior experience with other assessments. Twelve contributed to the WG2 and five to the WG3 reports. None was part of WG1, which includes fewer practitioners overall (Additional file 2: Table S2). The practitioners interviewed played a variety of roles, with 14 LAs—two playing an crucial role in “rescuing” a chapter that was struggling—and three CLAs. One of the three review editors described how undertaking this role allowed him to participate at all, as his external work responsibilities precluded his participation in the full process of drafting a chapter. Two interviewees participated in the AR5 scoping process, four in outreach activities, and three had other post-AR5 participation, including in the structured expert dialog, and making early career researchers aware of how they can engage with the IPCC.

#### Nomination experiences

Because the nomination process influences the diversity of authors of IPCC reports (Ho-Lem et al. 2011; Yamineva 2017), it is valuable to understand how this group of authors came to be involved in AR5. Of the 12 interviewees who described their nomination experiences, four were nominated by their country of citizenship, two by the country they worked in but were not citizens of, and one by an accredited observer organization. One of the practitioners nominated by their government described the following experience:

*“I was in a meeting with the Minister of Environment, and it was one of several presentations he was listening to. And at the end of it he said, this is the only presentation I understood, we need people like this in the IPCC” (Interviewee #14).*

Another practitioner was asked to submit her application after presenting at a conference, while a third asked for her name to be endorsed by her government focal point. Two were recruited after the assessment started to fill knowledge or expertise gaps, while a final practitioner was included as a LA after reviewing the first order draft. This person had not been nominated for either the fourth or fifth assessments (AR4 or AR5)—despite having expressed interest in participating to his employer, also an observer organization, and both countries he was a citizen of. After reviewing the first order draft, he was asked for further contributions, which gradually increased until he was included as a lead author of the chapter. Despite not being nominated, he contributed because:

*“There are certain things that I care about, how they go into the chapters, that make me continue to contribute” (Interviewee #5).*

#### Practitioner roles and contributions

Some of the roles and contributions of practitioner authors would be the same as those of academic authors: framing issues, drafting chapter sections, networking with authors across chapters, and/or subject expertise. However, most of the interviewed practitioners were able to work in both research and practice because they understood both worlds, and their key contributions related to this experience. These contributions highlight the value of these practitioners for ensuring the relevance of IPCC reports for end users, through their ability to identify on-the-ground policy needs. Moreover, their experience working with a wide range of stakeholders from different backgrounds provided them with the skills to communicate messages across worldviews and in a manner that is accessible to policymakers.

About half the interviewees identified the practical experience of how climate change issues are playing out in the real world as their most significant chapter contribution. For example:

*“There were times I heard sort of more academic discussion of things, and I would eventually lose patience and say, look, just stop. I’ve worked in how many bloody places, and that is not the case ... if you have been on the ground … and worked with people who are looking at you for solutions, you build the confidence to be able to do that … I don’t think academics can necessarily do that because they’re just in a different world” (Interviewee #1).*

Practical experience also informed another practitioner on what should be reflected in the report, despite the absence of peer-reviewed literature:

*“… there was a proliferation of stuff happening. A lot of it was at the pilot level, or developing of policies and not really implementing them well yet … And I was seeing this throughout Africa, and the literature didn’t reflect that ... [it] was extremely patchy and actually would have misrepresented the situation ... I knew that from … being in countries, doing the work, talking to other colleagues who were practitioners ... we were able to use grey literature to fill in some of the gaps. But I think you first had to know that, and then go and search for it” (Interviewee #12).*

Second, the reports are intended for a policy audience, and many of the interviewed practitioners were familiar with or were themselves end users. The following quote, by a Global South development bank employee, illustrates this perspective:

*“we work with government, we speak the language of decision makers ... And this is very important when we come to the key messages. So, you have to formulate the messages in the way that you speak to the policymakers. To put it in a form that it is evidence-based but also … understandable ... because at the end of the day the targets of [these] reports are policymakers. And if you don’t speak to them, what is the use of the report” (Interviewee #2).*

Another interviewee described their function as that of a translator, whose purpose was to translate from a different “language” to make information understandable to the end users, by simplifying academic language or introducing graphics to make concepts comprehensible.

Third, what some of the practitioner community brought was the ability to work across disciplines in an integrative way, as this interviewee conveyed:

*“I work with experts, but pull it together, structure it in a way, work across disciplines. My view is often putting it into a policy context, looking for the broader messages. That meant integrating across a lot of projections, a lot of different outcomes, and a lot of different disciplines, in a way that … was clear enough, simple enough to be understood by policymakers and the broader public” (Interviewee #6).*

This is a skill that is especially necessary to address climate change and other environmental crises around the world. Further specific contributions, related to these three key contributions, were the unlocking of new perspectives and interpersonal skills: bridging academic divides, balancing of strong points of view in a team, and networking across chapters.

#### Practitioners’ authoring experiences and challenges

The value of having practitioners as authors can only be realized if they are able to participate fully or actively (Yamineva 2017). In other words, if the aspects of the process weaken their contribution, then their presence on chapter teams will not improve the relevance of the reports for end users. We therefore describe the experiences of and main challenges faced by the interviewed practitioners during their time as IPCC authors, which segues neatly into a section describing suggestions raised by interview and survey respondents for the IPCC process to improve the usefulness of reports for end users.

Some experiences will be common to IPCC authors, whether academics or practitioners—such as the challenges of collaborative work where not everyone pulls their weight or the frustration of personally authored input not ending up in the final draft. Such experiences were identified by the interviewees, but practitioner-specific experiences have been highlighted here.

In terms of working with academics in chapter teams, most pointed to good working relations between the authors from these two communities and described being at ease working with academics, because of having worked in teams with academics before, or from working with academic literature. Only a few interviewees described experiences which highlighted the differences between the groups. The tendency of some to focus on the minutiae in the literature, which did not feel relevant to the discussion at the time, was echoed by another:

*“I did pick up on that schism between the pure academics – and not all academics are like that – but … where you made a comment [in plenary discussions and to authors of other chapters] that was related to real-world experience, it was sort of like, ‘well, that can’t be true because it’s not in the literature’” (Interviewee #12).*

Several interviewees pointed to the variation in the types of academics—one interviewee highlighted that some were “applied” academics, intent on making their research relevant.

Grey literature emerged as an issue relevant to the experience of practitioners, with four Global South interviewees flagging that published literature was not accurately reflecting reality in their subject area, and relevant grey literature was difficult to find:

*“That is why it is very difficult sometimes to find Africa-specific perspective[s] in these reports because of the lack of published material, especially coming to the level of the local experience ... Sometimes there are issues which you think are very important and wish to be part of the assessment process, but you don’t see any literature, so you just forget about it” (Interviewee #2).*

This is pertinent, because, with no evidence to cite, these perspectives on relevant issues may not be reflected. A practitioner from Africa described how, in the absence of the necessary literature, he and other LAs wrote a peer-reviewed paper that was then cited in the chapter. Another practitioner described how her stakeholder group expected more cases from practice to be included, yet there was *concern and hesitation* about using grey literature from some in the chapter team (interviewee #14), because this evidence was not written in an analytical manner. In the absence of grey literature, one practitioner was given the option of including relevant information in a box, while a Global North interviewee argued that the experience of writing was easier for academics because grey literature was not their supporting literature.

The challenge of finding time to contribute is faced both by academics and practitioners. However, practitioners face unique constraints in participation. One interviewee who was an academic when she participated in AR4, contrasted her participation experiences:

*“… when I was working as a researcher and a lecturer it was easier for me to participate in the IPCC process. There was more flexibility with my time … I viewed it like an academic exercise. Much easier than now, than coming from a development institute. My work at the university, some of these students help[ed] me with my research … so it was easier for me to work [as] a researcher and an academic in the IPCC process” (Interviewee #2).*

Many academics and researchers earn salaries from their institutions, and since such institutions recognize the prestige of participating in the IPCC, some paid time may be used to undertake IPCC-related work. This scenario differs with the circumstance of many practitioners, such as independent consultants, who are often only paid for hours worked and may not receive a fixed salary. Some had to forego income opportunities to participate:

*“In the end I turned down other work so that I could focus more on IPCC work. And I don’t think that that is properly recognized or valued. … I tried initially to get some funding … but it didn’t work, and I thought … I want to do this, I will do it, I will take the hit. I think it would put off a lot of people because they wouldn’t be able to do that ... or they would think, that’s unfair to expect me to fund myself through all of this, which is essentially what you are doing” (Interviewee #12).*

There were varying levels of support for practitioners who did have employers, some of whom allowed the use of paid work time for IPCC meetings, while others did not. Some practitioners took paid leave to attend meetings or spend time writing, with one fitting IPCC work in after her day job was over:

*“All of my IPCC work I did usually between 10pm and 2am, after I put the children to bed and after I finished the emails ... IPCC is extracurricular. Your day job is already so full” (Interviewee #8).*

### Suggestions by survey respondents and interviewees

Both survey and interview participants provided suggestions to facilitate practitioner involvement. These fall into two broad groups: improving the relevance and uptake of IPCC assessments for end users to inform action, as informed by practitioner experiences (Table 2) and improving the representation of practitioners in the assessments (Table 3). “More efficient assessment process” falls within both Tables 2 and 3, although the proposed actions have a different focus.

**Table 2:** Suggestions from study participants for improving the relevance and uptake of IPCC assessments, grouped into overarching themes

Improved accessibility and motivation for diverse end users	- Use simpler language and writing style and more graphics, particularly in the summary for policymakers, which needs to be relevant to and understandable by Global South policy/decision-makers - Present assessment findings as opportunities (innovation, economic, etc.) to get buy-in from audiences other than policymakers (e.g., industry) - Make clear the economic rationale behind acting against climate issues - Phrase motivation for change in a language/way that business people can understand
More flexible content and action-oriented focus	- Start with a broad plenary-approved outline, allowing more scope for development and refinement during the first lead author meeting (LAM) and alignment with other chapters to establish handovers and coherent storylines, gaps in content, and considering author team competences - Encourage and support wider disciplinary literature searches, including grey literature as a source of valuable public data and policy information, and the consideration of literature in different languages - Have more exchanges with policymakers of host countries during LAMs to get perspectives on their needs - Increase the emphasis on solutions, shifting the approach to include more of a practical focus on tackling climate change
More efficient assessment process	- Smaller, more frequent assessments of shorter duration so that the literature upon which the assessment is based is not outdated by the time the reports are published

**Table 3:** Suggestions from study participants on ways to enhance practitioner involvement in IPCC assessment processes

Make practitioners aware of	- Opportunity to participate as authors - Benefits of individual participation and for their organizations
Nomination	- Encourage more development institutions to be observer organizations (including from Global South) who can then nominate practitioners (and be more accommodating of their employees’ involvement as authors) - Advertise the nomination process among practitioner groups/in practitioner spaces (e.g., professional associations) - Obtain nominations by more than one government/observer organization to increase the chance of practitioner selection
Develop a deliberate strategy	- To identify potential practitioner candidates at the science-policy interface, working on more than local issues, with a wide network, and academic exposure (including from lending institutions) - To screen candidates once identified - To invest long-term in potential candidates who need preparation before involvement in future assessments - For a practitioner targeted induction - For chapters requiring a practitioner presence, specifying a desired ratio of academics to practitioners - To include practitioners in IPCC Bureau, scoping process, and outreach (for the latter, via practitioner networks)
More efficient assessment process	- Fewer LAMs - Better use of technology to facilitate remote teamwork and reduce travel - More facilitation by CLAs within chapter teams, who need to be explicit about input required by the lead authors - Provision of management coaching for CLAs, including inclusivity and transdisciplinary teams
Support practitioner authors unfamiliar with peer-reviewed literature	- Chapter scientist or research assistant support for literature searches
Possible non-author roles for practitioners	- Identifying key research gaps - Dialoguing instead of authoring - Providing real-world perspectives on proposed chapter recommendations

*IPCC* Intergovernmental Panel on Climate Change, *LAMs* lead author meetings, *CLAs* coordinating lead authors

## Discussion

In the contemporary world, the greatest challenges are multifaceted, cross-discipline, and require varied perspectives to find workable solutions (Hirsch Hadorn et al. 2006). Diverse teams working at the science-policy interface are best placed to identify solutions for these complex problems (Börner et al. 2010). While these teams should include scientists with considerable disciplinary depth, an overreliance on academic contributions may impair the translation of knowledge to practice, and result in a knowledge-implementation gap (Knight et al. 2008; Matzek et al. 2014; Weaver 2008).

Many of the skills identified as being necessary for effectively communicating research for policy audiences, or playing the role of an interpreter (Holmes and Savgård 2009, 716), or intermediary (Strydom et al. 2010), are exhibited by the sample of practitioners, or pracademics, interviewed in this study. Practitioners bring to the table a good understanding of the “intricacies of the political process,” which helps determine “what information needs to be transferred to policymakers, as well as how to package and present this information, … to improve the likelihood that it will be used” (Strydom et al. 2010, 4).

We verified that practitioners form a very small portion of IPCC authors, in AR5 and AR6, which varied by working group (WG1 had the fewest), and region, with practitioners making up a relatively large proportion of the African authors in AR5 when compared with other regions. Some factors potentially contributing to the relatively low proportion of African academics include high teaching loads, limited funding, and consequent lower research output (Beaudry et al. 2018; North et al. 2020), all of which contribute to the lower likelihood of these academics being nominated as—and agreeing to be—IPCC authors. The poorer representation of practitioners in WG1 is consistent with its focus on climate science, rather than more applied topics covered by the other two working groups, which require implementation and, therefore, benefit from input from authors with practical experience.

Beyond issues of representation, the survey respondents and interviewees noted distinct challenges impacting their participation: practitioners’ employers, or clients, frequently do not support their participation in extracurricular activities, such as IPCC work, to the same extent as academics may be supported (Posner 2009; Taylor et al. 2016). This includes the provision of time to work on IPCC tasks, which many practitioner authors had to fit into personal time, and the provision of income, requiring some practitioners to forego income when working on IPCC-related tasks. The authors based in “for-profit” organizations, driven and limited by human resources, time, and budget allocations, are restricted by the need to provide a commercial justification for their time (Howarth et al. 2017; Viner and Howarth 2014). Academic research is similar to authoring a global assessment, while for many practitioners, their everyday work contrasts sharply with this process (Rynes et al. 2001). Most of the respondents emphasized excessive time commitment, compounded by considerable travel, which limited their contribution. On this point, Victor (2015, 29) advises the IPCC to make more efficient use of volunteers’ time: “practically nothing else in science service has such a high ratio of input to output.” While the issue of travel is less relevant in AR6 due to COVID-19 restrictions, this research shows that, ultimately, practitioners had to balance working on the IPCC assessment with often-inflexible demands of their income-generating employment, something not necessarily compatible with IPCC needs, nor seen as having organizational value (Howarth et al. 2017).

Similar to the findings of Viner and Howarth (2014), some study participants noted that academic expertise was valued more highly than practitioner experience. However, few of the practitioners interviewed complained about working closely with the academic authors in their chapter teams. This may relate to their familiarity with working with diverse stakeholder groups, undertaking work that is academic in nature, or the advanced academic backgrounds of these participants. The practitioners participating in these assessments carry many of the hallmarks of pracademics (Posner 2009), with the ability to move fluidly between research and practice. It is possible, and probable, that practitioners with strong research backgrounds are more likely to make themselves available for these sorts of assessments, whereas practitioners unfamiliar with research are unlikely to do so.

Participants provided clear examples of the benefits of including practitioners in the IPCC process, which related to knowledge and experience of “on-the-ground” issues not adequately covered in the peer-reviewed literature, where practitioners were able to provide context and insight into what is feasible, or suggest alternative sources of information. This experience also allowed practitioners to bring the real-world context back into focus during chapter discussions.

Sometimes, the gap between practitioners’ experience and the assessed peer-reviewed literature could be bridged using grey or non-peer-reviewed literature. The IPCC has been hesitant about citing the grey literature (IPCC 2011, 3), preferring to focus on peer-reviewed literature to “increase the scientific legitimacy of the reports” (Devès et al. 2017, 146), after errors in AR4 jeopardized the organization’s reputation (Ravindranath 2010; Schiermeier 2010). While the grey literature may be cited, on the condition that the quality and validity of each source have been reviewed, and, if not publicly or commercially available, the document has been provided to the relevant TSU (IPCC 2010, 6-7, 2013, 17), interviewees emphasized that some specific, locally relevant knowledge gaps persist, particularly in countries of the Global South.

Practitioners’ goals tend to be pragmatic, community-oriented, and focused on solving problems, an approach that is at odds with the intentionally non-policy outlook of the IPCC (Stokols et al. 2008), which aims to provide information to policymakers without endorsing specific policies and to represent a credible and unbiased consensus (Schrope 2001). The intergovernmental assessment body walks a difficult line, since it synthesizes science that is highly germane to society, yet, for it to be continuously held as the gold standard to which other global environmental assessments aspire, it also needs to avoid politics in its product (Granjou et al. 2013). However, the IPCC does operate in a political context, and its reports have “far-reaching consequences for international politics,” which complicates practitioner participation (Beck and Mahony 2018; Schrope 2001, 112) and mean that it is likely to be cautious about practitioner influence in the SPM (Schrope 2001). Nevertheless, the urgency of the climate change challenge and the Paris Agreement’s call to action (United Nations 2015) point to the importance of it considering the contribution of this group of authors.

Moreover, with recent IPCC reports calling for urgent and deeper action to mitigate climate change and its impacts (IPCC 2018, 2021a), uptake and implementation of the reports’ findings are increasingly important. This requires decisions and action by policymakers, corporations, and individuals globally. The participants of this study had three key recommendations for improving uptake: improve accessibility for the intended users, including using appropriate language to engage diverse stakeholders; allow authors greater flexibility and control over chapter content, following the evidence and incorporating different sources of evidence to support locally-relevant, implementable solutions; and improve the efficiency of the process to ensure reports are timely, relevant, and based on the most recent literature.

It is critical that IPCC reports are written with end users in mind, if they are to make the most of the information presented. The language and writing style need to be accessible and appropriate, considering different terminologies used by different professions as well as non-scientific (or non-academic) audiences (Viner and Howarth 2014). The reports need to be structured to answer real-world questions and contain information that catches the attention of those responsible for enacting change (Howarth et al. 2017). Moreover, a support system is needed within governments to facilitate the use of the assessments, particularly in the Global South, including resources to appoint experts to advise politicians with the interpretation, choice, and implementation of the options presented.

With the outline of the reports currently determined during a scoping meeting of diverse experts before authors are selected (IPCC 2013), it leaves little room for adaptation by the authors based on their expertise, or the evolution of topics in the literature. The inclusion of diverse and locally relevant sources of evidence needs to be encouraged and supported if suitable solutions and adaptation strategies for climate change are to be identified (Ford et al. 2016; Kowarsch and Jabbour 2017). Yet, some Global South practitioner interviewees expressed frustration at not being able to include issues they know to be of relevance, because there may be no literature to reference on a particular topic. This highlights a need for additional research on emerging concerns in these contexts.

Overall, the literature on climate change is growing exponentially (Callaghan et al. 2020), making it challenging for IPCC authors to keep up to date. This, together with the IPCC’s cutoff dates for inclusion of the literature (IPCC 2010, 8), ultimately results in the assessment reports being out of date by the time they are published. Shorter, more targeted assessments may alleviate the time commitment and literature overload on authors, making it easier for practitioners to participate (Howarth et al. 2017).

One means of improving the relevance of the IPCC reports for target audiences would be to make sure practitioners are better incorporated into the process, both ensuring better inclusion and enabling active participation (Yamineva 2017).

The lack of author diversity has been largely attributed to a lack of diverse author nominations by governments at a procedural level (Ho-Lem et al. 2011; Yamineva 2017). A task group established to improve gender balance and address related issues within the IPCC during AR6 made recommendations to government representatives on this issue (IPCC Secretariat 2019), and this study highlights the need for such guidance relating to practitioners.

For global assessments to have a real impact, it is essential that they answer the questions of the intended users. Open communication between academics and practitioners, including openness to other systems of thought and to procedures that facilitate their active participation and influence during decision-making, is required (Cornell et al. 2013; Gordon et al. 2014). Integration requires active support (Mauser et al. 2013) and consideration of issues relating to the social contexts, practices, interests, motivations, and symbolic power of the authors involved (Hughes and Paterson 2017). The COVID-19 pandemic has meant that in-person global meetings have moved online, reducing travel, and compelling individuals and institutions to improve their online capacity. This provides an opportunity for globally dispersed experts, particularly practitioners, to participate in such assessments (Porpiglia et al. 2020). Unfortunately, transitioning online has also brought to light existing disparities in access to (sufficient) data and bandwidth, creating a different, and new set of problems for practitioners from the Global South, in particular (Treré 2021).

Rather than following a linear path from better evidence to better policy, knowledge and policy processes tend to form a “continuum of influence” (Holmes and Savgård 2009). In the short term, the benefits of involving practitioners as authors may include ensuring that the report is relevant to and ready for use by other practitioners and policymakers. In the longer term, a more subtle benefit of their inclusion may be the development of working relationships spanning practice and research communities, with the potential for bidirectional influence. Good relationships between these communities have been shown to be an important enabler of effective communication of scientific research (Holmes and Savgård 2009) and, in developing countries, can facilitate the uptake of scientific evidence into policy (Strydom et al. 2010).

While interviewees mentioned financial challenges to participation, none raised the issue of funding as something that should be considered to increase practitioner participation. One developing country author mentioned increasing the funding available, but as a means of increasing the participation of authors from developing countries, rather than specifically that of practitioners. Consequently, based on the results of this study, we cannot include increased funding as a recommendation. However, because it is possible that a lack of financial support would preclude some practitioners from participating entirely (and therefore not be included in the sample of IPCC authors surveyed by this study), we recommend future work to explore this issue further.

The coding of authors as practitioners was a major challenge for this study, despite the apparently clear-cut definition of Howarth et al. (2017). For example, since many authors transition between research and practice, we categorized them solely by their affiliation during the time they served as authors, to provide an indication of their work environments during the time spent contributing to the AR5 report. However, some practitioners undertake multiple types of work simultaneously, which may not be adequately represented by the affiliations reported by the IPCC, or on other web pages (e.g., LinkedIn, Google Scholar). Another challenge was that several potential practitioners had little to no online presence, making it very difficult to establish their employment and publication histories or obtain up-to-date contact information. Ideally, the identification of practitioners would be done after speaking with each author, to establish whether their day-to-day work was predominately applied or theoretical, an approach that was not feasible for this study. For future work, we recommend that the definition of practitioner be updated to account for these complexities, for example, basing the classification on an individual’s current work, rather than institutional affiliation. Furthermore, there should be greater distinction between practitioners with a great deal of academic experience (i.e., pracademics) and those without this experience, whose practical experience may not be best utilized as IPCC authors, but may provide context as reviewers, or during stakeholder consultations.

This study has documented the experiences of practitioners involved in the IPCC’s Fifth Assessment. It has not attempted to investigate the relationship between the inclusion of practitioners as authors of global assessments and the implementation or incorporation of recommendations into policy. This would be a highly relevant next step, for future research to attempt to untangle.

## Conclusions

In the climate change sphere, IPCC assessment reports inform international negotiations and the decisions of policy and practice communities. To ensure that the solution and adaptation options identified in IPCC reports meet the needs of their users, it is critical that authors with practical experience, as with all stakeholder groups, are adequately represented and able to participate actively (Beck et al. 2014; Yamineva 2017). Our findings indicate that practitioners are a valuable addition to IPCC author teams, improving the accessibility and relevance of IPCC assessments for decision-makers and thereby supporting climate action. By identifying challenges to their participation and describing possible solutions, we provide the IPCC with the opportunity to facilitate full participation and take advantage of the value this group of authors has to add. To improve practitioner representation and participation, the IPCC could learn from the Intergovernmental Panel on Biodiversity and Ecosystem Services, an organization which has grappled head-on with the issue of integration (Esguerra et al. 2017; Hotes and Opgenoorth 2014), by reviewing its own procedures, performance, and underlying assumptions, while navigating the challenge of “rebalancing scientific integrity and neutrality with political relevance and oversight” (Beck et al. 2014; Beck and Mahony 2018; Morin et al. 2017). This should be a critical first step undertaken at the start of the upcoming Seventh Assessment cycle.

## Supplementary Information

**Additional file 1.** Survey questions.

**Additional file 2: **Additional text and results. **Table S1.** Regional differences in the inclusion of practitioners as authors for each of the three IPCC AR5 Working Groups (WGs). **Table S2.** Diversity of authors of the Intergovernmental Panel on Climate Change’s Sixth Assessment Report (AR6), by Global North or South, region and gender, and practitioner status, and practitioners’ representation in different levels of chapter team leadership. **Table S3.** Comparing regional, gender, and practitioner representation, and practitioners involved in different levels of leadership, between the Fifth (AR5) and Sixth (AR6) Assessments of the Intergovernmental Panel on Climate Change. Underlying numbers are shown in Table S1. Cross-chapter papers were a new concept introduced into AR6, and thus are not relevant to AR5. **Figure S1.** Comparing regional representation among working groups for the fifth and sixth IPCC assessments, based on authors’ affiliations at the time of the assessment. **Figure S2.** Geographic distribution of respondents by birth, citizenship, education, and place of work. **Table S5.** Background information of the 29 practitioners who participated in this survey. **Table S6.** Survey respondents’ recommendations for improvement, grouped into overarching themes. **Table S7.** List of interviews and interviewees. **Table S8.** Details of interviewees at the time of their participation as authors of the Fifth Assessment of the Intergovernmental Panel on Climate Change.

## Data Availability

The datasets generated during the current study are not publicly available due to study participant confidentiality concerns but are available from the corresponding author upon reasonable request.
