# Supplementary material for: Science for implementation: the roles, experiences, and perceptions of practitioners involved in the Intergovernmental Panel on Climate Change
Source: Clim Action. 2022 Sep 24;1(1):25. doi: 10.1007/s44168-022-00025-2 (PMC9510420; doi:10.1007/s44168-022-00025-2)
Supplement: Supplementary file 1 — Additional file 1. Survey questions. [file 44168_2022_25_MOESM1_ESM.docx]

**Science for Implementation: The Roles, Experiences and Perceptions of Practitioners Involved in the Intergovernmental Panel on Climate Change**

North MA^a^*, Hunter NB^a^, Roberts DC^a,b^ and Slotow R^a,c^

^a^ School of Life Sciences, University of KwaZulu-Natal, Durban, South Africa

^b^ Sustainable and Resilient City Initiatives Unit, eThekwini Municipality, Durban, South Africa

^c^ Department of Genetics, Evolution and Environment, University College, London, UK

*Email addresses*: [ecotoxvet@gmail.com](mailto:ecotoxvet@gmail.com) (M. North) *, [huntern@ukzn.ac.za](mailto:huntern@ukzn.ac.za) (N. Hunter), [debra.roberts@durban.gov.za](mailto:debra.roberts@durban.gov.za) (D. Roberts), [Slotow@ukzn.ac.za](mailto:Slotow@ukzn.ac.za) (R. Slotow)

# Additional file 1: Survey Questions

**The purpose of this survey is to identify and understand the experiences of practitioners who have been involved in IPCC assessment processes in terms of both challenges and opportunities.**

**→ Before you start, please complete the informed consent form (page 2).**

**→ The questionnaire should take less than 30 minutes.**

**→ Please note that your responses to this survey are anonymous, all personal information will be kept for correspondence purposes only, and that you may withdraw your participation at any time.**

**Please complete all questions to the best of your abilities to ensure an accurate portrayal of the practitioner experience. Many of the questions provide limited either / or choices, however, some are open-ended to draw out your personal views on the answers. Please try to be as descriptive as possible.**

**For more information about this survey, please feel free to contact:**

**Removed for privacy concerns**

* 1. Please read the following and check the box below to acknowledge that you understand that:

The information that you provide will be used for scholarly research only.

Your participation is entirely voluntary. You have a choice to participate, not to participate, or to stop participating in the research. You will not be penalized for not participating.

Your responses to this survey will be presented anonymously. Neither your name nor identity will be disclosed in any form in the study.

The results of this survey will be held in a password-protected file accessible only to me and my supervisor. After a period of 5 years, in line with the rules of the university, all digital records will be deleted.

I understand

* 2. **DECLARATION**:

I hereby confirm that I understand the contents of this document and the nature of the research project "Practitioner Perspectives of the Intergovernmental Panel on Climate Change (IPCC)".

I understand that I am at liberty to withdraw from the project at any time, should I so desire. I understand the intention of the research. I hereby agree to participate.

With my participation, I hereby provide consent for the information obtained in this survey to be used for scholarly research and publication in peer-reviewed journals.

I understand the purpose and procedures of the study and have been given an opportunity to answer questions about the study and have had answers to my satisfaction.

By checking this box, I hereby acknowledge that I understand all the points above and agree to participate in this study.

- 3. If you have any further questions/concerns or queries related to the study, you understand that you may contact the researcher at [removed for privacy concerns]. If you have any questions or concerns about your rights as a study participant, or if you are concerned about an aspect of the study or the researchers then you may contact:

Removed for privacy concerns.

I have seen this information

- 1. Please indicate which of the following best describes you:


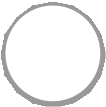
 Male
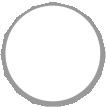
 Female


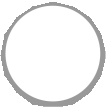
 I choose not to answer

- 1. What is your country of birth?
  2. What is your country of citizenship?
  3.
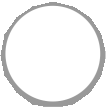
What is your highest level of education?


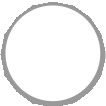

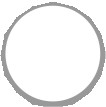
 Diploma
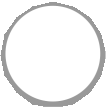
 Bachelors
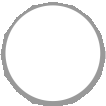
 Honours

Other (please specify)


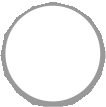
 Masters Doctoral (PhD)

Demographics & Occupational Background

- 1. Where did you get the qualification listed in the previous question?

Name of the academic institution:

Country:

- 1. In which country are you currently employed?
  2. What language(s) do you speak at home?
  3. What is your preferred language for work?
  4. What are your areas of expertise? Select all that apply.

Climatology Meteorology Modelling Biology Ecology Botany

Agriculture (livestock) Agriculture (plants) Forestry

Entomology Microbiology Virology

Vector-borne disease Parasitology

Food security Risk Assessment

Physics Chemistry Policy Communication Urban design Geography Geology Energy

Remote sensing Oceanography/oceanology Marine biology Engineering

Health sciences/medicine Glaciology

Economics Other

- 1. If your area(s) of expertise was not listed above, please list below:
  2. In which sector(s) are you currently employed? Check all that apply.

Public (Government) Private (Industry)

Non-Governmental Organisation (NGO) Academic

Other (please specify)

- 1. In which field(s) are you employed? Check all that apply.

Research Consulting Policy Construction Media

Public Relations Energy

Conservation & Ecology Transport

Planning & Development Environmental Management Local Government Provincial Government National Government

Other (please specify)

- 1. How long have you been involved in the work that got you involved in the IPCC process? Please round to the nearest year.
  2. In what capacity(ies) have you been involved in IPCC processes? Select all that apply.

Co-chair

Coordinating Lead Author Lead Author

Review Editor

Contributing Author Chapter Scientist Expert Reviewer

Other (please specify)

- 1.
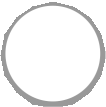
Would you be interested in participating in future IPCC assessment cycles?


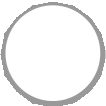

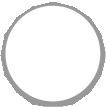
 Yes No

Maybe

Could you please explain why you answered what you did?

- 1. When considering your most recent experience (AR5 or previous) working with the IPCC, how would you rate the following statements?


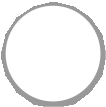

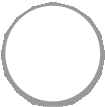

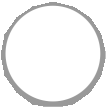

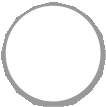

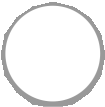
Strongly Agree Agree Disagree Strongly Disagree N/A


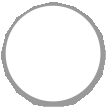

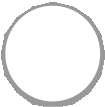

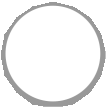

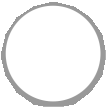

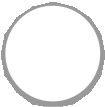


There is a culture of inclusivity among IPCC authors

I felt as though the co- chairs of my working group tried to include everyone


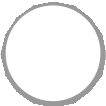

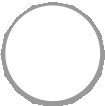

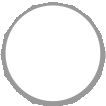

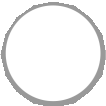

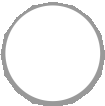


I felt as though my input during the assessment was valued


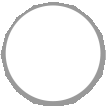

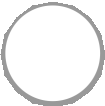

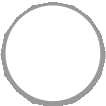

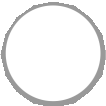

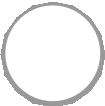
Working with the IPCC provided valuable opportunities for networking


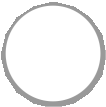

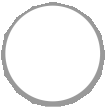

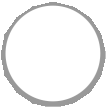

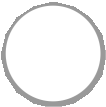

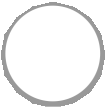


Working with the IPCC has benefitted my career

My employer understood and

supported my
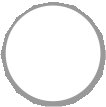

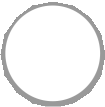

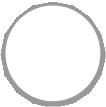

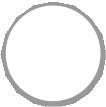

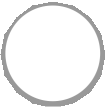


commitment to the IPCC


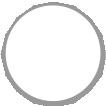

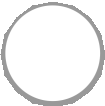

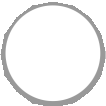

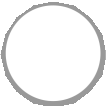

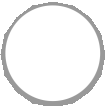


The collaboration between authors from the global north and global south is mutually beneficial

Authors from all countries have equal

influence on the
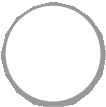

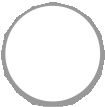

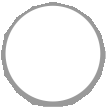

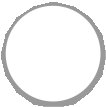

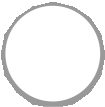


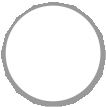

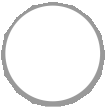

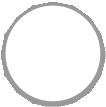

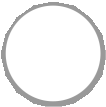

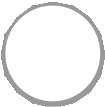
content of the final report


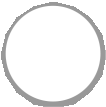

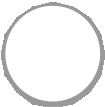

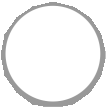

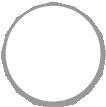

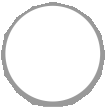


My experience as a practitioner was valued as highly as academic scientific expertise

My experience of policy implementation was valued as highly as academic scientific expertise


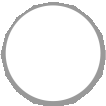

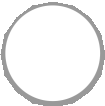

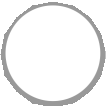

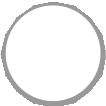

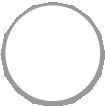


I feel that the IPCC assessment

process is equitable for all genders

Strongly Agree Agree Disagree Strongly Disagree N/A

I feel that in the IPCC, everyone is valued for

what they can
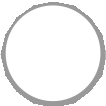

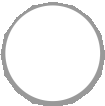

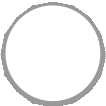

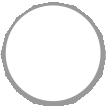

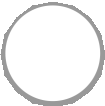


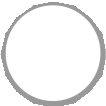

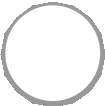

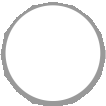

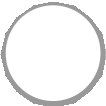

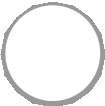
contribute regardless of their gender


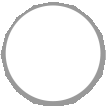

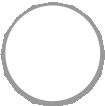

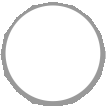

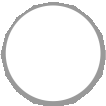

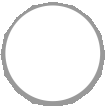


I felt discriminated against at some time during my time working with the IPCC

I felt that the other authors in my chapter supported me throughout the process


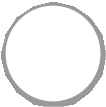

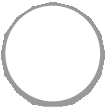

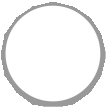

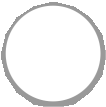

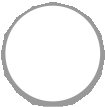


I felt that the coordinating lead authors supported me throughout the process


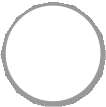

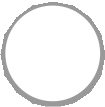

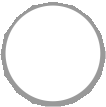

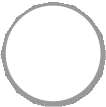

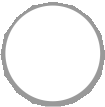
I felt that my contributions were equitably included in the final report


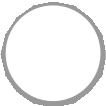

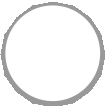

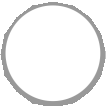

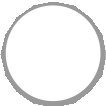

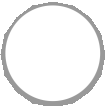


The workload was divided fairly among authors within my chapter

- 1. When it comes to authorship of IPCC reports, please rank how important you think the following are for ensuring the best product (1 - highest importance, 5 - lowest importance):


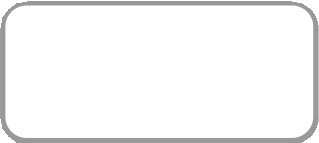

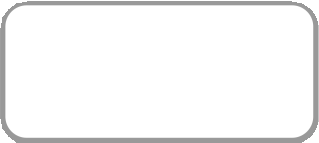

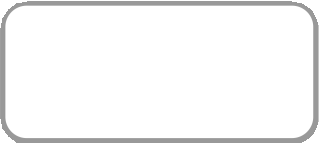


Scientific expertise

Policy experience

Gender equality

Regional representation

Ethnic diversity

- 1. Please rank the following to indicate your perception of author influence on the content of IPCC reports (1 - highest influence to 3 - lowest influence):

Authors from academic institutions

Authors working for local or higher levels of government (public sector)

Authors in industry (private sector)

- 1. Regarding your experience of working with the IPCC: Please select how much you agree or disagree with the statements below.

Strongly Agree Agree Disagree Strongly Disagree N/A

Required excessive time commitment

There was sufficient administrative support

Excessive travel was required

There was a power

imbalance within my chapter team

I experienced interpersonal conflict within my team

There was poor

communication within my team

There was poor communication among teams

I received inadequate support from my Government Focal Point

I received inadequate support from my employer

My productivity was impacted by limited or inadequate internet access

My productivity was impacted by limited access to literature

The deadlines set by

the IPCC were unreasonable

- 1. Please rate your overall personal experience of working with the IPCC.

Positive Neutral

Negative

- 1. Are there any aspects of the IPCC assessment processes that you would change?

Yes No

If yes, please specify which aspects you would like changed.

Your perception of how others experienced the IPCC processes

- 1. How do you think other authors experienced working with the IPCC?

I feel that my experience of the process reflects the average author experience Most authors had a better experience than I did

Most authors had a worse experience than I did I am not sure

- 1. Compare your experiences with chapter authorship to your impression of the experiences of authors from other chapters:

Better than most About average Worse than most N/A

Team cohesion

Interpersonal conflicts

Chapter organisation

Supportive leadership

Dealing with diversity (ethnicity, gender, language, etc.)

- 1. Please describe any other impediments to your productivity as an author of an IPCC report, or any other problem areas in general that have not been touched upon in this survey.
