# Supplementary material for: Science for implementation: the roles, experiences, and perceptions of practitioners involved in the Intergovernmental Panel on Climate Change
Source: Clim Action. 2022 Sep 24;1(1):25. doi: 10.1007/s44168-022-00025-2 (PMC9510420; doi:10.1007/s44168-022-00025-2)
Supplement: Supplementary file 2 — Additional file 2: Additional text and results. Table S1. Regional differences in the inclusion of practitioners as authors for each of the three IPCC AR5 Working Groups (WGs). Table S2. Diversity of authors of the Intergovernmental Panel on Climate Change’s Sixth Assessment Report (AR6), by Global North or South, region and gender, and practitioner status, and practitioners’ representation in different levels of chapter team leadership. Table S3. Comparing regional, gender, and practitioner representation, and practitioners involved in different levels of leadership, between the Fifth (AR5) and Sixth (AR6) Assessments of the Intergovernmental Panel on Climate Change. Underlying numbers are shown in Table S1. Cross-chapter papers were a new concept introduced into AR6, and thus are not relevant to AR5. Figure S1. Comparing regional representation among working groups for the fifth and sixth IPCC assessments, based on authors’ affiliations at the time of the assessment. Figure S2. Geographic distribution of respondents by birth, citizenship, education, and place of work. Table S5. Background information of the 29 practitioners who participated in this survey. Table S6. Survey respondents’ recommendations for improvement, grouped into overarching themes. Table S7. List of interviews and interviewees. Table S8. Details of interviewees at the time of their participation as authors of the Fifth Assessment of the Intergovernmental Panel on Climate Change. [file 44168_2022_25_MOESM2_ESM.docx]

**Science for Implementation: The Roles, Experiences and Perceptions of Practitioners Involved in the Intergovernmental Panel on Climate Change**

North MA^a^*, Hunter NB^a^, Roberts DC^a,b^ and Slotow R^a,c^

^a^ School of Life Sciences, University of KwaZulu-Natal, Durban, South Africa

^b^ Sustainable and Resilient City Initiatives Unit, eThekwini Municipality, Durban, South Africa

^c^ Department of Genetics, Evolution and Environment, University College, London, UK

*Email addresses*: [ecotoxvet@gmail.com](mailto:ecotoxvet@gmail.com) (M. North) *, [huntern@ukzn.ac.za](mailto:huntern@ukzn.ac.za) (N. Hunter), [debra.roberts@durban.gov.za](mailto:debra.roberts@durban.gov.za) (D. Roberts), [Slotow@ukzn.ac.za](mailto:Slotow@ukzn.ac.za) (R. Slotow)

# Additional file 2: Additional text and results

## Methods Detail

Three rounds of fortnightly email reminders were sent by the lead researcher, followed by a single reminder four months later, with the study co-authors copied, to maximise the response rate. The survey (see Additional file 1) starts with a brief background with contact information, followed by compulsory informed consent questions confirming that the participants understand and acknowledge that this survey is for research purposes. The first 13 questions cover the demographic, educational, and occupational background of the respondents, the next eight questions are about their experience of the IPCC process, followed by two ‘outward’ looking questions about their perceptions of other people’s experiences of the IPCC, and a final, open-ended question which asks participants to describe any issues not covered by any of the previous questions. Most of the questions are multiple-choice or short answer questions to maximise response rate. Questions 19 and 22 include statements for which participants must indicate how strongly they disagree or agree; no neutral option was provided to prevent respondents from consistently choosing the neutral option. Seven respondents completed the survey after the initial request, two more participated after the first reminder, and four after the second reminder; after the final email, four more participants completed the survey.

After an initial round of revision, reviewers requested a modification of focus (beyond African practitioners only), and an expansion of the data collection to incorporate more participants and generate deeper interrogation of the issues. This was conducted in 2021, with requests for further surveys emailed to the larger pool of practitioners as well as requests for interviews. Ultimately, 29 practitioners completed the survey, and 17 the interview.

## Results

### AR5 authors

**Table S1.** Regional differences in the inclusion of practitioners as authors for each of the three IPCC AR5 Working Groups (WGs) (region for one WG2 author unknown)

| Working Group | Region | Total | % of total | Practitioner | | Other | |
| --- | --- | --- | --- | --- | --- | --- | --- |
|  |  |  |  | n | % | n | % |
| WG1 | Africa | 12 | 5% | 4 | 33% | 8 | 67% |
|  | Asia | 44 | 17% | 1 | 2% | 43 | 98% |
|  | Europe | 100 | 39% | 0 | 0% | 100 | 100% |
|  | Latin America and the Caribbean | 10 | 4% | 0 | 0% | 10 | 100% |
|  | North America | 71 | 28% | 1 | 1% | 70 | 99% |
|  | Oceania | 18 | 7% | 0 | 0% | 18 | 100% |
|  | ***Total*** | ***255*** |  | ***6*** | ***2%*** | ***249*** | ***98%*** |
| WG2 | Africa | 34 | 12% | 13 | 38% | 21 | 62% |
|  | Asia | 44 | 15% | 7 | 16% | 37 | 84% |
|  | Europe | 89 | 31% | 6 | 7% | 83 | 93% |
|  | Latin America and the Caribbean | 31 | 11% | 2 | 6% | 29 | 94% |
|  | North America | 66 | 23% | 5 | 8% | 61 | 92% |
|  | Oceania | 26 | 9% | 5 | 19% | 21 | 81% |
|  | *Unknown* | *1* | 0% | *1* | 100% |  | 0% |
|  | ***Total*** | ***291*** |  | ***39*** | ***13%*** | ***252*** | ***87%*** |
| WG3 | Africa | 26 | 10% | 14 | 54% | 12 | 46% |
|  | Asia | 55 | 20% | 6 | 11% | 49 | 89% |
|  | Europe | 89 | 33% | 10 | 11% | 79 | 89% |
|  | Latin America and the Caribbean | 35 | 13% | 7 | 20% | 28 | 80% |
|  | North America | 57 | 21% | 6 | 11% | 51 | 89% |
|  | Oceania | 10 | 4% | 2 | 20% | 8 | 80% |
|  | ***Total*** | ***272*** |  | ***45*** | ***17%*** | ***227*** | ***83%*** |

Comparing AR5 and AR6

Table S2 contains the same information as Table 1, but for AR6 rather than AR5.

**Table S2.** Diversity of authors of the Intergovernmental Panel on Climate Change’s Sixth Assessment Report (AR6), by Global North or South, region and gender, and practitioner status, and practitioners’ representation in different levels of chapter team leadership

|  |  |  |  | Practitioner | | Other | |
| --- | --- | --- | --- | --- | --- | --- | --- |
|  |  | Total | % of total | n | % | n | % |
| Socio-economic division | North | 517 | 65% | 46 | 9% | 471 | 91% |
|  | South | 279 | 35% | 49 | 18% | 230 | 82% |
| Region | Africa | 70 | 9% | 17 | 24% | 53 | 76% |
|  | Asia | 169 | 21% | 21 | 12% | 148 | 88% |
|  | Europe | 291 | 37% | 31 | 11% | 260 | 89% |
|  | Latin America and the Caribbean | 78 | 10% | 11 | 14% | 67 | 86% |
|  | North America | 115 | 14% | 7 | 6% | 108 | 94% |
|  | Oceania | 73 | 9% | 8 | 11% | 65 | 89% |
| Gender | Women | 273 | 34% | 30 | 11% | 243 | 89% |
|  | Men | 523 | 66% | 65 | 12% | 458 | 88% |
| Leadership roles | Coordinating lead author (CLA) | 114 | 14% | 10 | 9% | 104 | 91% |
|  | Lead author (LA) | 513 | 64% | 54 | 11% | 459 | 89% |
|  | Review editor (RE) | 117 | 15% | 26 | 22% | 91 | 78% |
|  | Cross-chapter paper lead | 15 | 2% | - | 0% | 15 | 100% |
|  | Cross-chapter paper author | 37 | 5% | 5 | 14% | 32 | 86% |
|  | Grand Total | 796 |  | 95 | 12% | 701 | 88% |

**Table S3.** Comparing regional, gender, and practitioner representation, and practitioners involved in different levels of leadership, between the Fifth (AR5) and Sixth (AR6) Assessments of the Intergovernmental Panel on Climate Change. Underlying numbers are shown in the Supplementary Information (SI Table S1). Cross-chapter papers were a new concept introduced into AR6, and thus are not relevant to AR5.

|  |  | Practitioner (%)^‡^ | |
| --- | --- | --- | --- |
|  |  | AR5 | AR6 |
| Socio-economic division | North | *6* | *9* |
|  | South | *22* | *18* |
| Region | Africa | *43* | *24* |
|  | Asia | *10* | *12* |
|  | Europe | *6* | *11* |
|  | Latin America and the Caribbean | *12* | *14* |
|  | North America | *6* | *6* |
|  | Oceania | *13* | *11* |
| Gender | Women | *13* | *11* |
|  | Men | *10* | *12* |
| Role | Coordinating Lead Author (CLA) | *9* | *9* |
|  | Lead Author (LA) | *11* | *11* |
|  | Review Editor (RE) | *12* | *22* |
|  | Cross-chapter paper lead | *-* | *0* |
|  | Cross-chapter paper author | *-* | *14* |
|  | Grand Total (%) | *11* | *12* |

^‡^ Only the percentage of practitioners is shown here for each category, for AR5 and AR6. The percentage of non-practitioners are the values reported in Tables S1 and S2.


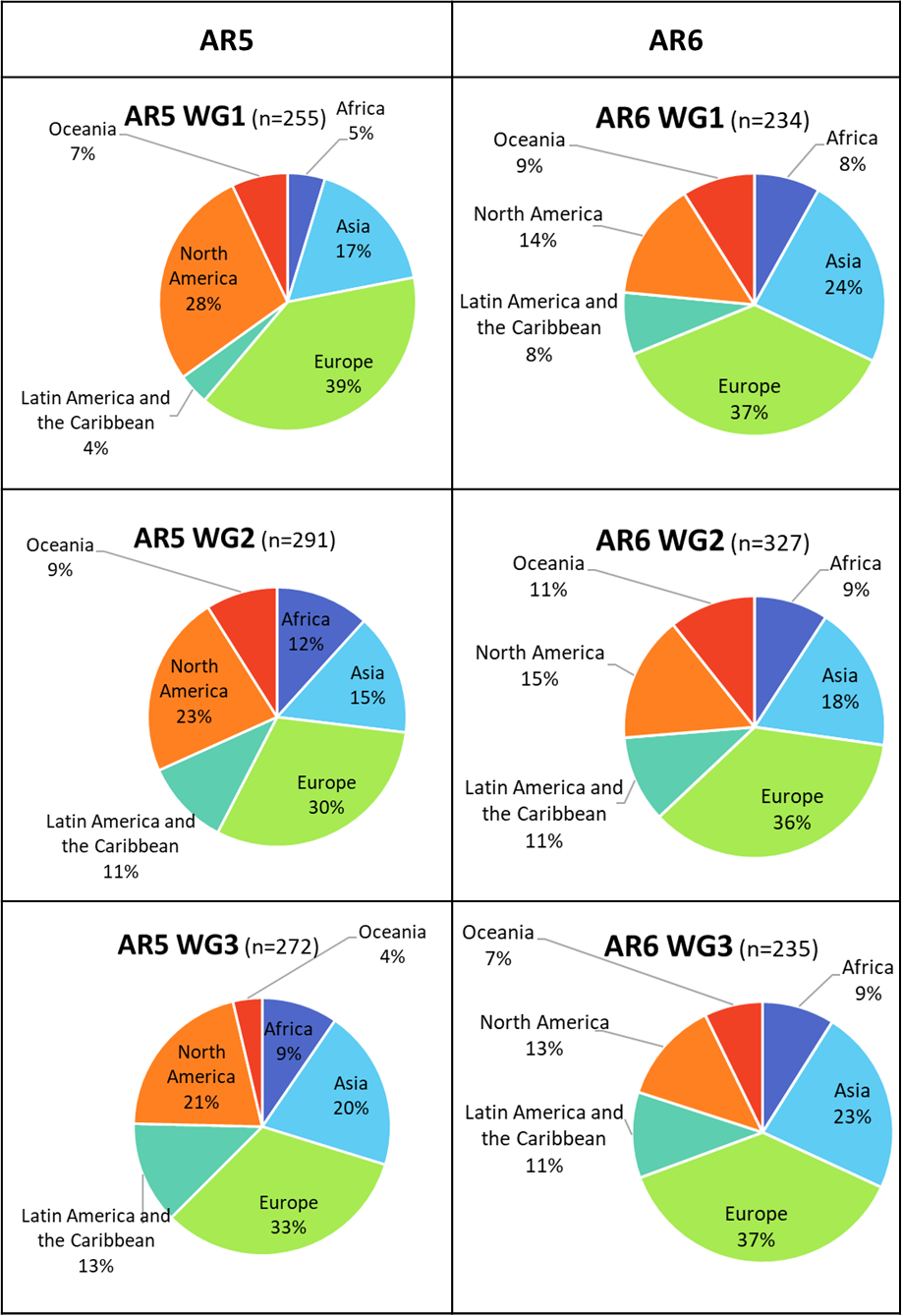


**Figure S1.** Comparing regional representation among working groups for the fifth and sixth IPCC assessments, based on authors’ affiliations at the time of the assessment.

### Background information: survey respondents

Of the 29 respondents, ten identified as women and 19 as men. They represented 21 nationalities, mainly African or European, and all but one had postgraduate education (23 PhDs and five master’s degrees as highest qualification; unspecified for one participant), with degrees conferred from 28 institutions in 15 countries (almost half from the USA (7), UK (4) or South Africa (3)) (Figure S2). The respondents preferred to communicate in several languages, with English most mentioned for home (19 respondents) and work (27). Ten respondents were multi-lingual, speaking two or more languages at home, with three comfortable using two or three languages for work.


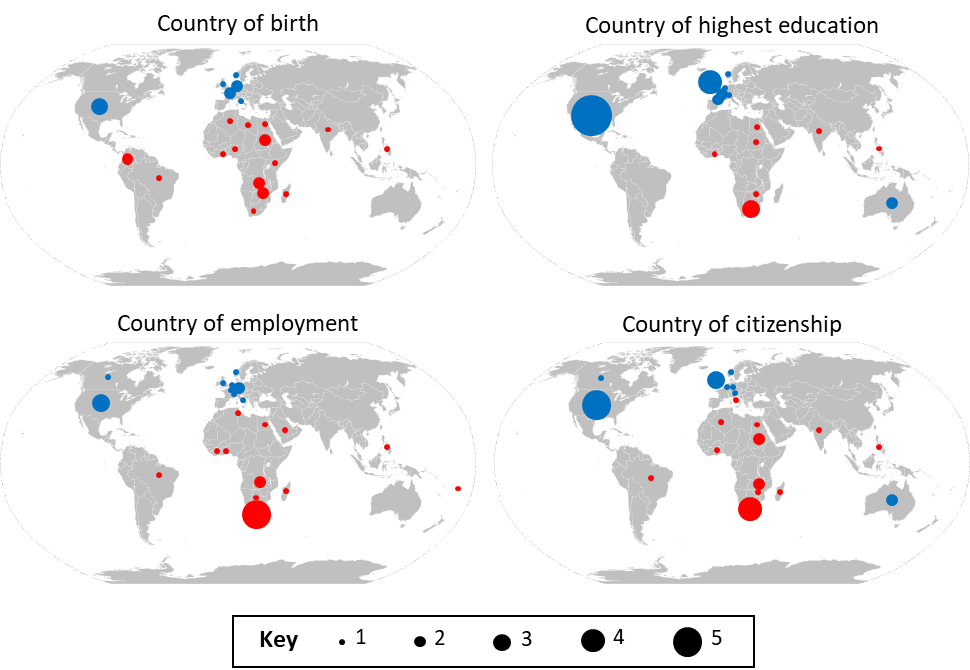


**Figure S2.** Geographic distribution of respondents by birth, citizenship, education, and place of work. Maps modified from public domain images from Wikimedia commons. Dots are colored based on whether they are in the Global North (blue) or South (red).

The respondents were experts in diverse disciplines, including climatology, ecology, food security, and urban design, with 16 reporting expertise in policy. Most worked in the public sector (eight), private sector or for NGOs (six each); however, many engaged across sectors and fields of work, with most reporting involvement in policy (19), research (18) and consulting (15). Respondents had an average of 20 years of experience in their field (range: 8–42 years). They had been variously involved in all aspects of the IPCC process: from technical administration (two served as CSs), authoring (eight CAs, 25 LAs and five CLAs), reviewing or editing the reports (11 ERs and eight REs), to levels of management in the IPCC, including a WG Co-Chair and WG Vice-Chair (further detail on this background information in Supplementary Information, Table S5).

The women had, on average, more experience working in climate change-related fields than men (20 years vs. 15 years), and all women had participated in IPCC assessments in two or more roles (average of three, including Expert Reviewer, Contributing Author, Lead Author, Coordinating Lead Author, Co-Chair, and Vice-Chair), whereas six of the eleven men had participated in only one role, either as a Review Editor (two) or Lead Author (four).

**Table S5.** Background information of the 29 practitioners who participated in this survey

| **Background** | **Results (number of respondents)** | | |
| --- | --- | --- | --- |
| Gender | Female (10) | | Male (19) |
| Education | Doctoral (23)  Masters (5) | | Other (1) |
| Home language(s)* | **Unilingual (19)**:  English (11)  Arabic (3)  Malagasy (1)  Spanish (1)  Portuguese (1)  Norwegian (1)  Dutch (1) | | **Multilingual (10):**  English and   - Akan (1) - local Zambian dialects (1) - Gilbertese (1) - Filipino (1) - Italian (1) - Bangla and Hindi (1) - German and Spanish (1) - Sesotho and isiZulu (basic) (1)   French and Arabic (1)  German and Spanish (1) |
| Preferred language(s) for work* | **Unilingual (26):**  English (24)  French (1)  Norwegian (1) | | **Multilingual (3):**  Arabic and English (1)  English and German (1)  English, German and Spanish (1) |
| Current areas of expertise* | Policy (16)  Energy (12)  Risk Assessment (11)  Economics (7)  Climatology (6)  Ecology (5)  Food security (5)  Geography (4)  Meteorology (4)  Modelling (4)  Biology (3)  Agriculture (crops) (2)  Botany (2)  Chemistry (2)  Engineering (2)  Forestry (2)  Urban Design (2) | | Agriculture (livestock) (1)  Climate Finance (1)  Climate Change Adaptation (1)  Climate Change Mitigation (1)  Climate and Health (1)  Climate Strategy and Programme Development (1)  Development Studies (1)  Futures Research (1)  Geology (1)  Oceanography (1)  Physics (1)  Remote Sensing (1)  Strategic Planning (1)  Virology (1) |
| Current sector* | Public (Government) (8)  Private (Industry) (6)  Non-Governmental Organisation (NGO) (6)  Independent/Private Consultant/Practitioner (4) | | Academic (4) **  Multilateral Development Bank (2)  State-Owned Entity (1) |
| Current field of employment* | Policy (19)  Research (18)  Consulting (15)  Energy (11)  Environmental Management (9)  Planning & Development (9)  Local Government (5) | | National Government (4)  Conservation & Ecology (3)  International Organisation (2)  Transport (2)  United Nations (2)  Public Relations (1) |
| Roles in the IPCC | Vice-chair (1)  Co-chair (1)  Coordinating lead author (5)  Lead author (25) | | Review editor (8)  Contributing author (8)  Chapter scientist (2)  Expert reviewer (11) |
| Years of experience in this field | 5-9: 3  10-14: 7  15-19: 2 | 20-24: 9  30-34: 7  >40: 1 | |

* Does not add up to 29 because several participants listed multiple languages, and participants were able to check off as many areas of expertise, sectors and fields of employment as were relevant to them.

** All four respondents who checked off ‘academic’ also listed other sectors (NGO, private (industry), other)

For the next two sections, the information is only for the first round of surveys that included African practitioners, and is no longer presented in the main manuscript.

African practitioners only: Survey responses

When asked whether the participants would be interested in participating in future assessments with the IPCC, twelve said that they would, two said they might, and three said that they would not. Those not interested in participating described not having sufficient time; wanting to allow new people to lead the process; how the lack of congruence with day-to-day work made it hard to accommodate both; and that the “…*process is [not] set up to facilitate the involvement of independent policy practitioners*”.

Participants who wanted to participate again cited: their interest in new topics being covered; the largest scientific co-production project … critical for global sustainability; the opportunity for knowledge sharing and working with international experts; it being highly rewarding. One respondent described it as “*a good opportunity to contribute, as [an] African, to delivering … useful scientific information for … large … stakeholders/communities*”.

Interestingly, practitioners ranked scientific expertise as the characteristic most important for authoring IPCC reports, followed by policy experience, regional representation, gender equality, and ethnic diversity. Authors from academic institutions were perceived to have the highest influence on content, followed by public sector authors, with private sector authors perceived as having the lowest influence. Women and men ranked these criteria equally.

### African practitioners only: Survey respondents’ recommendations

Eleven of the seventeen respondents said there were aspects of the IPCC assessment process they would change, and thirteen provided examples (see Table SI.S6).

Table S6. Survey respondents’ recommendations for improvement, grouped into overarching themes

| Aspect | Recommendations |
| --- | --- |
| IPCC assessment process | Conduct continuous assessments that are updated in real time to keep up to date with the increasing body of literature  Make better use of modern technology to facilitate remote teamwork and reduce required travel  Improve author selection process  Introduce targeted induction to help practitioners understand IPCC processes and jargon: importance of practitioners repeatedly emphasized by the IPCC, but practitioners not sufficiently accommodated  Develop a method of deterring authors who receive the benefits of being listed on the reports, but do not do the work assigned to them  Manage conflict of interest better, including mechanisms for authors to report perceived incidents of conflict of interest |
| Diversity | Continue working towards more diverse participation  Improve involvement of nonacademic authors  Make regional representation more equal |
| Developing country representation | Include more experts from developing countries as authors  Increase African representation at all stages, including during expert review of draft reports  Incentivize participation for authors from developing countries, for example by payment for time spent on the IPCC report |
| Developing country influence | Improve developing world perspective – including through situating IPCC power hubs (e.g., TSUs) within developing countries  Ensure developing country policy objectives are addressed  Summary for Policymakers should be relevant to and understandable by developing country policy/decision makers  Improve author access to literature on, and from, developing countries |

### Background information: interviews and interviewees

Table S7. List of interviews and interviewees

| **Number** | **Date** | **Length** | **Employment position during AR5** | **Institution** |
| --- | --- | --- | --- | --- |
| 1 | 28-May-21 | 0:51:10 | Technical advisor | Intergovernmental |
| 2 | 07-Apr-21 | 01:01:25 | Specialist | Development bank |
| 3 | 01-Apr-21 | 00:43:13 | Consultant | Consultancy |
| 4 | 18-Mar-21 | 00:57:38 | Director | Government |
| 5 | 28-May-21 | 00:51:35 | Senior officer | Intergovernmental |
| 6 | 22-Apr-21 | 01:09:16 | Consultant | Consultancy |
| 7 | 14-May-21 | 00:45:40 | Consultant | Consultancy |
| 8 | 20-May-21 | 00:43:38 | Head & executive director | Intergovernmental & NGO |
| 9 | 17-May-21 | 00:54:35 | Consultant & head of science | Consultancy & NGO |
| 10 | 14-May-21 | 00:55:08 | Director, then consultant | NGO, then consultancy |
| 11 | 31-May-21 | 01:04:02 | CEO | Private company |
| 12 | 09-Mar-21 | 00:57:44 | Consultant | Consultancy |
| 13 | 18-May-21 | 00:45:24 | Consultant & research fellow | Consultancy & teaching and research |
| 14 | 04-May-21 | 00:56:23 | Senior advisor | NGO |
| 15 | 21-May-21 | 01:08:53 | Consultant, then government employee, then consultant | Consultancy, then government, then consultancy |
| 16 | 25-Mar-21 | 01:01:21 | Consultant | Consultancy |
| 17 | 06-Apr-21 | 00:31:55 | Division chief | Development bank |

**Table S8**. Details of interviewees at the time of their participation as authors of the Fifth Assessment of the Intergovernmental Panel on Climate Change

| **#** | **Job description** | **Continent of citizenship** | **GN/ GS (citizen-ship)** | **Gender** | **Highest qualification** | **# pubs ResearchGate* (h-index)** | **Working Group** | **Author roles in AR5**† | **# chapters pre-AR5 (LA/CLA/RE)** | **# chapters in AR5** | **Nr of chapters in AR6** | **Organization** | | | | | | |
| --- | --- | --- | --- | --- | --- | --- | --- | --- | --- | --- | --- | --- | --- | --- | --- | --- | --- | --- |
|  |  |  |  |  |  |  |  |  |  |  |  | **Pvt co** | **Consultancy** | **Research** | **Intergov** | **Dev Bank** | **NGO** | **Gov** |
| 1 | Technical advisor | Australasia | North | Male | PhD | 21 (12) | WG2 | LA | 0 | 1 | 0 |  |  |  | 1 |  |  |  |
| 2 | Specialist | Africa | South | Female | PhD | 46 (17) | WG2 | LA & CLA | 1 | 2 | 1 |  |  |  |  | 1 |  |  |
| 3 | Consultant | North America | North | Male | PhD | 80 (22) | WG3 | LA | 3 | 1 | 1 |  | 1 |  |  |  |  |  |
| 4 | Director | Africa | South | Male | PhD | n/a | WG3 | LA | 0 | 1 | 0 |  |  |  |  |  |  | 1 |
| 5 | Senior officer | Europe | North | Male | PhD | 198 (49) | WG3 | LA | 1 | 1 | 1 |  |  |  | 1 |  |  |  |
| 6 | Consultant | North America | North | Male | Masters | 102 (40) | WG2 | CLA | 5 | 1 | 0 |  | 1 |  |  |  |  |  |
| 7 | Consultant | Europe | North | Female | PhD | 97 (27) | WG2 | LA | 0 | 1 | 0 |  | 1 |  |  |  |  |  |
| 8 | Head & executive director | North America | North | Female | PhD | 97 (26) | WG2 | LA | 0 | 1 | 0 |  |  |  | 1 |  | 1 |  |
| 9 | Consultant & head of science | Europe | North | Male | PhD | 84 (30) | WG2 | CLA & LA | 0 | 3 | 1 |  | 1 |  |  |  | 1 |  |
| 10 | Director, then consultant | Africa | South | Male | PhD | n/a | WG2 | RE | 0 | 1 | 0 |  | 1 |  |  |  | 1 |  |
| 11 | CEO | North America | North | Male | Masters | n/a | WG3 | LA | 0 | 1 | 0 | 1 |  |  |  |  |  |  |
| 12 | Consultant | Africa | South | Female | Masters | n/a | WG2 | LA | 0 | 1 | ‡ |  | 1 |  |  |  |  |  |
| 13 | Consultant & research associate | Asia | South | Female | PhD | n/a | WG2 | RE & LA | 1 | 2 | 2 |  | 1 | 1 |  |  |  |  |
| 14 | Senior advisor | Asia | South | Female | Other | n/a | WG2 | LA | 0 | 1 | 1 |  |  |  |  |  | 1 |  |
| 15 | Consultant, then state employee, then consultant | Australasia | North | Female | PhD | n/a | WG2 | LA | 0 | 1 | 0 |  | 1 |  |  |  |  | 1 |
| 16 | Consultant | Africa | South | Male | PhD | n/a | WG3 | LA | 0 | 2 | 1 |  | 1 |  |  |  |  |  |
| 17 | Division chief | North & South America | North | Male | Masters | 64 (18) | WG2 | RE | 0 | 1 | 0 |  |  |  |  | 1 |  |  |

* some authors did not have a ResearchGate profile, numbers of publications both grey and peer-reviewed publications listed; † roles in AR5 include both main and special reports; ‡ participated as Lead Author but resigned; Pvt co = private company; Intergov = intergovernmental organization; Dev Bank = development bank; Gov = government
